# Women with multiple gestations have an increased risk of development of hypertension in the future

**DOI:** 10.1186/s12884-021-03992-2

**Published:** 2021-07-16

**Authors:** Geum Joon Cho, Un Suk Jung, Ho Yeon Kim, Soo Bin Lee, Minjeong Kim, Ki-Hoon Ahn, Sung Won Han, Soon-Cheol Hong, Hai-Joong Kim, Younghan Kim, Min-Jeong Oh

**Affiliations:** 1 Department, of Obstetrics and Gynecology, Korea University College of Medicine, Seoul, Republic of Korea; 2 Department of Obstetrics and Gynecology, Hanyang University Guri Hospital, College of Medicine, Hanyang University, Guri-si, Republic of Korea; 3 Deparment of Obstetrics and Gynecology, Severance Hospital, Yonsei University Health System, Seoul, Republic of Korea; 4 School of Industrial Management Engineering, Korea University, Seoul, Republic of Korea

**Keywords:** Multiple gestation, Hypertension, Preeclampsia

## Abstract

**Background:**

Multiple gestations are associated with an increased incidence of preeclampsia. However, there exists no evidence for an association between multiple gestations and development of hypertension(HTN) later in life. This study aimed to determine whether multiple gestations are associated with HTN beyond the peripartum period.

**Methods:**

In this retrospective nationwide population-based study, women who delivered a baby between January 1, 2007, and December 31, 2008, and underwent a national health screening examination within one year prior to their pregnancy were included. Subsequently, we tracked the occurrence of HTN during follow-up until December 31, 2015, using International Classification of Diseases-10th Revision codes.

**Results:**

Among 362,821 women who gave birth during the study period, 4,944 (1.36%) women had multiple gestations. The cumulative incidence of HTN was higher in multiple gestations group compared with singleton group (5.95% vs. 3.78%, *p* < 0.01, respectively). On the Cox proportional hazards models, the risk of HTN was increased in women with multiple gestations (HR 1.35, 95% CI 1.19, 1.54) compared with those with singleton after adjustment for age, primiparity, preeclampsia, atrial fibrillation, body mass index, blood pressure, diabetes mellitus, high total cholesterol, abnormal liver function test, regular exercise, and smoking status.

**Conclusions:**

Multiple gestations are associated with an increased risk of HTN later in life. Therefore, guidelines for the management of high-risk patients after delivery should be established.

## Introduction

Growing evidence indicates that women affected by pregnancy complications, including preeclampsia, gestational diabetes, and preterm deliveries have an increased risk for the development of cardiovascular disease later in life [1–3]. Thus, targeted monitoring and management for women with pregnancy complications are recommended to decrease the rates of cardiovascular diseases (CVD) after delivery [4, 5].

Recent decades have seen a major increase in multiple gestations rates globally [6, 7]. In the US, twin births continue to rise from 1.9% in 1980 to 3.3% of all live births in 2009 [6]. Multiple gestations are associated with a significant increase in the risk of perinatal and neonatal morbidity and mortality, especially the ones associated with prematurity [8]. Moreover, this condition is usually considered as a risk for the mother as well. Women with multiple gestations have an increased risk of pregnancy complications including preeclampsia and gestational diabetes during pregnancy [9]. Several studies also reported the increased risk of CVD such as cardiomyopathy, heart failure, and myocardial infarction, but these studies investigated the effects of multiple gestations on the development of CVD only during peripartum period [10–12]. However, there exist limited data regarding the long-term effect of multiple gestations on the development of CVD, especially, the development of hypertension (HTN) later in. Accordingly, this study aimed to determine whether multiple gestations are associated with HTN beyond the peripartum period.

## Methods

### Characteristics of the data

This study was conducted by merging the databases of the Korea National Health Insurance (KNHI) claims and National Health Screening Examination (NHSE).

**Fig. 1:**
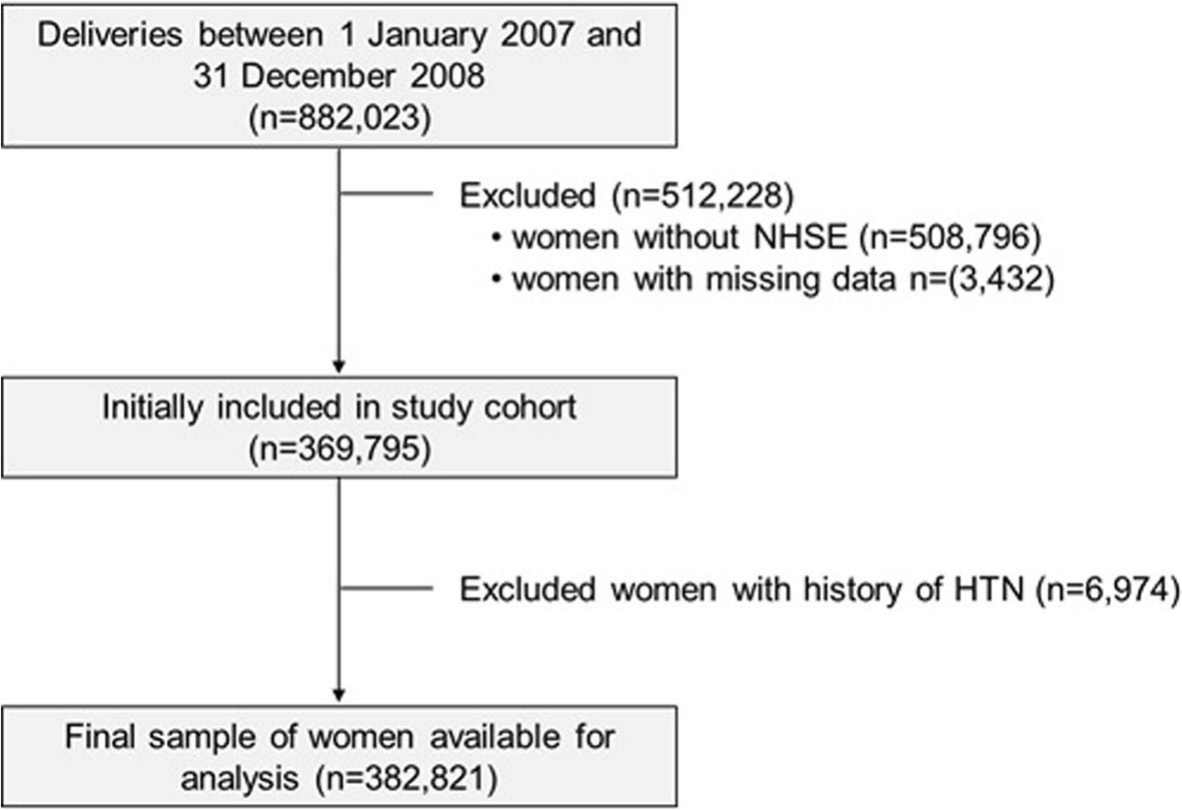
The enrollment of study participants

In Korea, 97% of the population participates in the KNHI program, and the KNHI claims that the database includes all medical information for each person except medical information not covered by insurance such as cosmetics. Using the KNHI claims database, all women who delivered between January 1, 2007, and December 31, 2008 were enrolled. A flowchart of patient enrollment is shown in Fig. 1. To facilitate the evaluation of the pre-pregnancy characteristics, women were included in the analysis if they underwent an NHSE within one year prior to their pregnancy. Among them, women with pre-pregnancy HTN were excluded.

Subsequent development of HTN was tracked until December 31, 2015.

Enrollees in the KNHI system were invited to undergo a standardized NHSE. The pre-pregnancy characteristics of our study population were reviewed from the NHSE data. The NHSE contains two parts: a health interview and examination. This study was approved by the Institutional Review Boards of the Korea University Medical Center (2018GR0401) and this committee granted an exemption from requiring informed consent.

### Study outcomes

Using the KNHI claims dataset, women with preeclampsia during their pregnancy were identified by a principal or secondary diagnosis based on the International Classification of Diseases-10th Revision (ICD-10). Women were classified as having HTN if they were newly diagnosed with HTN (ICD-10 code, I10-I15) from the date of delivery to December 31, 2015. The timing of the initial diagnosis was confirmed by the lack of a medical claim for HTN as a primary or secondary diagnosis before pregnancy.

### Measurements and definition

Based on the KNHI claims dataset, pregnancy factors including multiple pregnancies, the delivery mode and preeclampsia were confirmed.

Pre-pregnancy factors were evaluated using the KNHI claims dataset and the NHSE data. Pre-pregnancy diagnosis of atrial fibrillation was identified using ICD-10 code (I48) on the KNHI claims dataset. Smoking status and exercise were identified using health questionnaires on the NHSE data. Regular exercise was defined as moderate-intensity exercise performed at least 3 d/wk.

The health examination included the calculation of body mass index ([BMI] in kg/m^2^) using height and weight measurements. Obesity was defined as BMI ≥ 25 kg/m^2^. Blood pressure (BP) was measured using a standard mercury sphygmomanometer. Women with systolic BP (SBP) ≥ 140 and/or diastolic BP (DBP) ≥ 90 mmHg were excluded from the study. Blood samples were obtained after a fast of at least 8 h. The levels of fasting glucose, total cholesterol (TC), aspartate aminotransferase (AST), and alanine aminotransferase (ALT) were measured. Fasting glucose ≥ 126 mg/dL at the NHSE or a medical claim for DM as a primary or secondary diagnosis before pregnancy based on ICD-10 codes (E10-E14) defined diabetes mellitus (DM). High TC was defined as TC ≥ 200 mg/dL and abnormal liver function test was defined as AST ≥ 31 or ALT ≥ 31 mg/dL.

### Statistical analysis

Clinical and biochemical characteristics were compared among groups using the *t*-test for continuous variables and the χ^2^ test for categorical variables, expressed as mean ± SD and percentages, respectively. The cumulative incidence of HTN was estimated using the Kaplan–Meier method and compared using the log-rank test. We also determined the incidence rate of HTN (person-years). Cox proportional hazards models were used to estimate the adjusted hazard ratios (HRs) and 95% confidence intervals (CIs) for the development of HTN. Participants were censored if they developed HTN or on December 31, 2015, in those without HTN. All tests were two-sided, and p-values < 0.05 were considered statistically significant. Statistical analyses were performed using SAS for Windows, version 9.4 (SAS Inc., Cary, NC, USA).

## Results

Among 362,821 women who gave birth during the study period and underwent an NHSE within one year prior to their pregnancy, 4,944 (1.36%) women had multiple gestations.

### Characteristics of participants with respect to the number of pregnancies

The pre-pregnancy and pregnancy characteristics of participants are presented in Table 1. Women with multiple gestations were older and had a higher prevalence of advanced age, primiparity and preeclampsia compared with women with singleton pregnancy.

**Table 1:** The pre-pregnancy and pregnancy characteristics of participants stratified by the number of pregnancies

	Singleton (*n* = 357,877)	Multiple gestations (*n* = 4,944)	*P*-value
Age (years)	29.95 ± 3.27	30.02 ± 3.38	< 0.01
Advanced maternal age (%)	30,899 (8.63)	685 (13.86)	< 0.01
Primiparity (%)	214,451 (59.92)	3,893 (78.74)	< 0.01
Preeclampsia (%)	5919 (1.65)	374 (7.56)	< 0.01
Atrial fibrillation (%)	176 (0.05)	3 (0.06)	0.72
Regular exercise (%)	27,520 (7.69)	420 (8.50)	0.04
Smoker (%)	9,859 (2.75)	108 (2.18)	0.01
BMI (kg/m^2^)	20.75 ± 5.81	20.85 ± 2.71	0.23
Obesity (%)	23,335 (6.52)	371 (7.50)	< 0.01
Systolic BP (mmHg)	110.4 ± 10.54	110.6 ± 10.77	0.08
Diastolic BP (mmHg)	69.52 ± 7.86	69.60 ± 7.94	0.50
Fasting glucose (mg/dL)	85.93 ± 14.42	86.34 ± 12.87	0.04
DM (%)	3,324 (0.93)	71 (1.44)	< 0.01
TC (mg/dL)	174.0 ± 34.56	175.1 ± 35.20	0.03
High TC (%)	63,302 (17.69)	923 (18.67)	0.07
AST (mg/dL)	19.53 ± 11.38	19.89 ± 10.70	0.03
ALT (mg/dL)	15.41 ± 15.48	15.81 ± 14.27	0.07
Abnormal LFT (%)	13,531 (3.78)	205 (4.15)	0.18

Regular exercise was defined as moderate-intensity exercise performed at least 3 d/wk

Obesity by BMI; BMI ≥ 25 kg/m^2^, DM; fasting glucose ≥ 126 mg/dL at the NHSE or a medical claim for DM as a primary or secondary diagnosis before pregnancy based on ICD-10 codes, high TC; TC ≥ 200 mg/Dl, abnormal LFT; AST ≥ 31 or ALT ≥ 31 mg/dL

Values are expressed as mean (SD) or n(%)

*BMI* body mass index, *BP* blood pressure, *DM* diabetes mellitus, *TC* total cholesterol, *AST* aspartate aminotransferase, *ALT* alanine aminotransferase, *LFT* liver function test

Women with multiple gestations had a higher level of fasting glucose, TC, AST, the prevalence of obesity, and DM and a lower prevalence of smoking but higher prevalence of regular exercise compared with women with singleton pregnancy. However, there were no differences in BMI, BP, ALT levels, and the prevalence of atrial fibrillation, high TC and abnormal LFT between the two groups.

### Risk of HTN with respect to the number of pregnancies

**Fig. 2:**
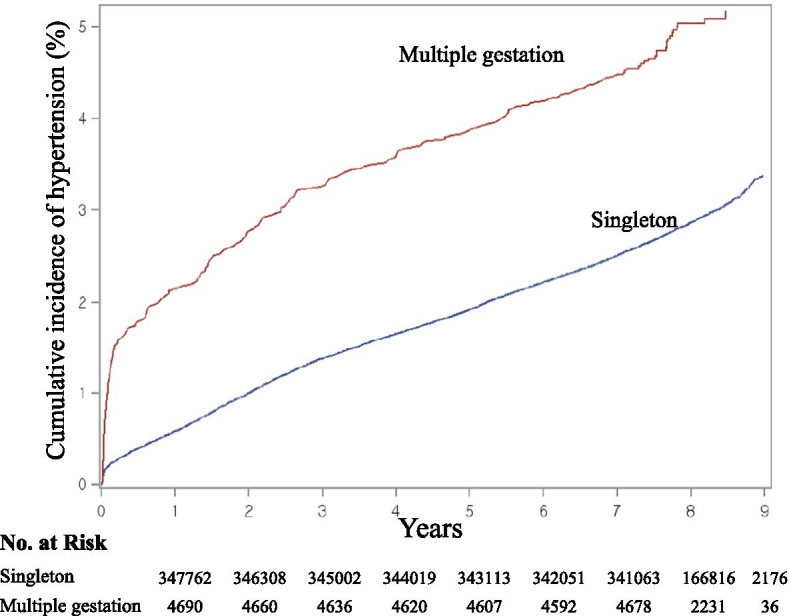
The Kaplan–Meier curves for the cumulative incidence of HTN between singleton and multiple gestations groups

Figure 2 shows the Kaplan–Meier curves for the cumulative incidence of HTN between singleton and multiple gestations groups. During follow-up, the cumulative incidence of HTN was higher in multiple gestations group compared with singleton group (5.95% vs. 3.78%, *p* < *0.01*, respectively).

Cox proportional hazards models were employed to estimate the adjusted HRs and 95% CIs for the development of HTN with respect to the number of pregnancies (Table 2). Increased risk of HTN was observed in women with multiple gestations (HR 1.35, 95% CI 1.19, 1.54) compared with those with a singleton after adjustment for age, primiparity, preeclampsia, atrial fibrillation, BMI, BP, DM, high TC, abnormal LFT, regular exercise, and smoking status.

**Table 2:** Risk of the development of HTN

	Incidence rate (per 1,000 person-years)	Unadjusted HR (95% CI)	Adjusted HR^a^ (95% CI)
Singleton	3.69	1	1
Multiple gestations	6.44	1.74 (1.53, 1.98)	1.35 (1.19, 1.54)

^a^Adjusted for age, primiparity, preeclampsia, atrial fibrillation, BMI, BP, DM, high TC, abnormal LFT, regular exercise and smoking status

Cox proportional hazards models were created to estimate the adjusted HRs for the development of HTN with respect to the number of pregnancies and the presence of preeclampsia (Table 3). According to the proportional hazards models, the risks of HTN were significantly increased in women with singleton and preeclampsia (HR 6.87, 95% CI 6.42, 7.35), women with multiple gestations but no preeclampsia (HR 1.49, 95% CI 1.28, 1.74) and women with multiple gestations and preeclampsia (HR 7.37, 95% CI 5.75, 9.45) compared with women with singleton but no preeclampsia after adjustment for age, primiparity, preeclampsia, atrial fibrillation, BMI, BP, DM, high TC, abnormal LFT, regular exercise, and smoking status.

**Table 3:** Risk of the development of HTN

	Incidence rate (per 1,000 person-years)	Unadjusted HR (95% CI)	Adjusted HR^a^ (95% CI)
Singleton without preeclampsia	3.36	1	1
Singleton with preeclampsia	27.27	7.95 (7.45, 8.48)	6.87 (6.42, 7.35)
Multiple gestations without preeclampsia	5.02	1.49 (1.28, 1.73)	1.49 (1.28, 1.74)
Multiple gestations with preeclampsia	27.24	7.98 (6.23, 10.20)	7.37 (5.75, 9.45)

^a^Adjusted for age, primiparity, preeclampsia, atrial fibrillation, BMI, BP, DM, high TC, abnormal LFT, regular exercise and smoking status

## Discussion

### Main finding

In this study, we evaluated the association between multiple gestations and the development of HTN after delivery and found that women with multiple gestations had an increased risk of HTN during 7 years follow-up period compared with those with singleton. In addition, as risk ratios for HTN seem to be additive, it was noted that a woman with multiple gestations combined with preeclampsia was at seven-fold increased risk of HTN. It is well-established that the risk of preeclampsia is greater in multiple gestations rather than in singleton pregnancies [9]. Preeclampsia is thought in part to explain the physiological mechanism by which the association between multiple gestations and the development of HTN is mediated, since women who have had preeclampsia have a high risk of ultimately developing HTN, later in life [13]. However, in this study, multiple gestations remained significantly associated with the development of HTN later in life when preeclampsia was adjusted in multivariable analysis or participants were divided based on the presence of preeclampsia, suggesting the existence of another mechanism.

### Strength and limitation

Our findings should be interpreted with caution due to several limitations. First, we were unable to access information regarding the characteristics of multiple gestations. It has been reported that the rate of severe preeclampsia was significantly increased in triplet pregnancy as compared to twin pregnancy although there was no change in the overall rate of preeclampsia [14]. In addition, several studies reported that the association of preeclampsia was different by chorionicity [15, 16] although others were inconclusive [17]. Thus, it is hypothesized that the association of multiple gestations with the development of HTN may be affected by the number and chorionicity of multiple gestations. Based on the results of the present study, further studies are necessitated to confirm these associations considering these factors. Second, in this study, the subjects were limited to women who had undergone the NHSE before pregnancy to adjust the pre-pregnancy factors for HTN. However, when we analyzed data of all pregnant women who delivered during the study period regardless of pre-pregnancy characteristics (869,822 of singleton and 12,201 of multiple gestations), similar association of multiple gestations with the development of HTN in later life after adjustment for age, primiparity and preeclampsia (HR 1.21, 95% CI 1.19, 1.30) was observed. Third, there were several other confounding factors not adjusted such as fertility treatment which is closely related to maternal old age, multiple gestations, HTN and other obstetric complications. In addition, women who had multiple gestations before 2007 and had subsequent singleton pregnancy were not considered as history of multiple gestation. The KNHI databases does not contain detailed previous obstetric histories and chorionicity in each patient. Last, the results of our study are not generalizable because the enrolled population enrolled was East Asian and our health system might be different from other countries.

Nevertheless, the strength of the present study is that this is the first study to evaluate the association between multiple gestations and the development of HTN later in life after adjustment for the pre-pregnancy factors for HTN with a large population-based long-term follow-up.

### Interpretation

Several potential explanations for these associations are possible. First, physiological changes related to multiple gestations during pregnancy may be directly attributed to the development of HTN. In multiple gestations, pronounced hemodynamic changes including greater cardiac output, development of left ventricular mass, fractional shortening and ejection fraction, and lower total vascular resistance develop compared to singleton pregnancy possibly due to the unique physiological changes required to meet the demands of a growing fetus [18, 19]. Consequently, in uncomplicated twin gestations, significant changes in systolic and diastolic function occur from the first to the third trimester mimicking a diastolic dysfunction as observed in the early stages of chronic cardiac insufficiency [20]. Moreover, although diastolic parameters normalize after pregnancy, a relative systolic dysfunction seems to persist after delivery [20]. These findings are similar with those shown at postpartum echocardiography in women who developed a preterm or severe preeclampsia in a singleton pregnancy [21, 22]. Thus, it is possible that the workload to the heart by the increased circulating volumes in multiple gestations may still affect cardiovascular systems leading to the development of HTN in later life. Moreover, in a twin pregnancy, circulating levels of antiangiogenic substances such as sFlt-1 and sEng [18], which have a pivotal role in the pathogenesis of the maternal syndrome in preeclampsia [19], were increased owing to large placental volume. Consequently, it is hypothesized that endothelial dysfunction caused by increased levels of antiangiogenic factors may cause HTN later in life even if preeclampsia did not occur during pregnancy.

Second, this association may be due to the particular characteristics of women with multiple gestations. An accumulating body of research has shown that numerous pregnancy complications appear to be preceded by subclinical vascular and metabolic dysfunction [23–26], suggesting that the complications during pregnancy may be useful markers of latent high-risk CVD [27]. In the present study, women with multiple gestations had a high incidence of cardiovascular risk such as obesity and DM, which are well-known risk factors for HTN. Thus, the increased risk of HTN later in life may be attributed to common predisposing factors for both multiple gestations and risk of HTN while it remains uncertain whether multiple gestations exacerbates previously unrecognized risk factors of HTN, or if the risk for HTN is the direct result of the manifestation of the disorder itself. Moreover, it has been known that maternal weight gain increases with increasing number of fetuses [28]. Given that excessive gestational weight gain may increase of the risk for the development of cardiovascular and metabolic disease through long-term maternal abdominal adiposity [29], women with multiple gestations may have an increased risk for the development of HTN later in life.

Lastly, it is well known that the rate of multiple gestations has increased owing to the expanded use of assisted reproduction [30, 31]. Previously studies reported the link between fertility treatments with pregnancy complications including preeclampsia [32–35]. Therefore, it is hypothesized that fertility treatments may attribute to the development of HTN in cases of multiple gestations. However, multiple gestations are suggested to be responsible for a large proportion of pregnancy complications associated with fertility [36]. However, as the data on fertility treatments were not available during the course of the present study, studies with these factors are warranted in the future to confirm our findings.

Guidelines for the management of multiple gestations have previously been published with information about pre-pregnancy and pregnancy management for multiple gestations to minimize the pregnancy complication related to multiple gestations [36, 37], but there is a lack of established guidelines or consensus regarding long-term follow-up. Based on our results, women with multiple gestations are considered as the high-risk group for the development of HTN later in life.

## Conclusions

Women with multiple gestations have an increased risk of HTN later in life. Therefore, guidelines for the management of these high-risk patients should be established. Women with existing cardiovascular disease such as HTN should be warned about consequences of multiple gestations in short and longterm. The results should be provided to physicians managing multiple gestations to aid in the process of patient counseling regarding the associated risks. However there have been no definitive preventive strategies for HTN so far, all women especially who had previous multiple gestations should be given lifestyle and dietary advice and monitored for the development of HTN.

## Data Availability

The dataset generated during and analyzed during the current study are not publicly available due to dataset owned by government but are available from the corresponding author on reasonable request.

## Abbreviations

KNHI: Korea National Health Insurance claims
NHSE: National Health Screening Examination
HTN: Hypertension
DM: Diabetes mellitus
CVD: Cardiovascular disease
BMI: Body mass index
BP: Blood pressure
AST: Aspartate aminotransferase
ALT: Alanine aminotransferase
LFT: Liver function test
