# Correction to: Women with multiple gestations have an increased risk of development of hypertension in the future

**DOI:** 10.1186/s12884-021-04120-w

**Published:** 2021-09-28

**Authors:** Geum Joon Cho, Un Suk Jung, Ho Yeon Kim, Soo Bin Lee, Minjeong Kim, Ki-Hoon Ahn, Sung Won Han, Soon-Cheol Hong, Hai-Joong Kim, Younghan Kim, Min-Jeong Oh

**Affiliations:** 1 Department of Obstetrics and Gynecology, Korea University College of Medicine, Seoul, Republic of Korea; 2 Department of Obstetrics and Gynecology, Hanyang University Guri Hospital, College of Medicine, Hanyang University, Seoul, Republic of Korea; 3 School of Industrial Management Engineering, Korea University, Seoul, Republic of Korea; 4 Deparment of Obstetrics and Gynecology, Severance Hospital, Yonsei University Health System, Seoul, Republic of Korea

**Correction to: BMC Pregnancy Childbirth 21, 510 (2021)**

**https://doi.org/10.1186/s12884-021-03992-2**

Following publication of the original article [1], it was noticed that the affiliation of authors were incorrect. The affiliations of each authors are as follows:

Geum Joon Cho ^1^†; Un Suk Jung ^2^†; Ho Yeon Kim ^1^; Soo Bin Lee^3^; Minjeong Kim ^1^; Ki-Hoon Ahn ^1^; Sung Won Han ^3^; Soon-Cheol Hong^1^; Hai-Joong Kim ^1^; Younghan Kim ^4^*†; Min-Jeong Oh ^1^*†

^1^Department of Obstetrics and Gynecology, Korea University College of Medicine, Seoul, Republic of Korea

^2^Department of Obstetrics and Gynecology, Hanyang University Guri Hospital, College of Medicine, Hanyang University, Seoul, Republic of Korea

^3^School of Industrial Management Engineering, Korea University, Seoul, Republic of Korea

^4^Deparment of Obstetrics and Gynecology, Severance Hospital, Yonsei University Health System, Seoul, Republic of Korea

The original article [1] has been updated.
